# The Role of Surface and Underlying Forms When Processing Tonal Alternations in Mandarin Chinese: A Mismatch Negativity Study

**DOI:** 10.3389/fpsyg.2020.00646

**Published:** 2020-04-08

**Authors:** Yu-Fu Chien, Xiao Yang, Robert Fiorentino, Joan A. Sereno

**Affiliations:** ^1^Department of Chinese Language and Literature, Fudan University, Shanghai, China; ^2^Department of Modern Languages, DePaul University, Chicago, IL, United States; ^3^Department of Linguistics, University of Kansas, Lawrence, KS, United States

**Keywords:** event-related potentials, mismatch negativity, tone, tone 3 sandhi, phonological alternation, Mandarin Chinese, spoken word recognition, lexical representation

## Abstract

Phonological alternation (sound change depending on the phonological environment) poses challenges to spoken word recognition models. Mandarin Chinese T3 sandhi is such a phenomenon in which a tone 3 (T3) changes into a tone 2 (T2) when followed by another T3. In a mismatch negativity (MMN) study examining Mandarin Chinese T3 sandhi, participants passively listened to either a T2 word [tʂu2 je4] /tʂu2 je4/, a T3 word [tʂu3 je4] /tʂu3 je4/, a sandhi word [tʂu2 jen3] /tʂu3 jen3/, or a mix of T3 and sandhi word standards. The deviant in each condition was a T2 word [tʂu2]. Results showed an MMN only in the T2 and T3 conditions but not in the Sandhi or Mix conditions. All conditions also yielded omission MMNs. This pattern cannot be explained based on the surface forms of standards and deviants; rather these data suggest an underspecified or underlying T3 stored linguistic representation used in spoken word processing.

## Introduction

Speech is variable. The acoustic characteristics of phonemes, words, and sentences change due to a variety of factors including speaking rate, speaking style, talker, and phonological environment (Cheng, 1966; Goldinger et al., 1991). Recognition of spoken words thus requires mapping the variable speech input onto stored lexical representations. Understanding how the brain represents these lexical categories is relevant for theories of speech perception, as theories differ in terms of the abstract nature of the mental representations as well as the role that surface acoustic and underlying linguistic representations play in speech processing.

The phonological environment affects the acoustic realization of segments in different contexts (e.g., Reetz and Jongman, 2009). In American English, for example, the phoneme /t/ is voiceless aspirated [t^h^] in initial position of a stressed syllable (as in “top”), voiceless unaspirated [t] when preceded by a sibilant (as in “stop”), and a voiced alveolar flap [ɾ] between a stressed and an unstressed vowel (as in “matter”). The phonological environment also influences the phonetic realization of tones in tone languages (Wang and Li, 1967). For instance, a Mandarin Chinese tone 3 syllable (a low-dipping tone) changes into a tone 2 syllable (a high-rising tone) when followed by another tone 3 syllable, a phenomenon known as Mandarin Chinese tone 3 sandhi. Given such phonological alternations, a key question arises as to how listeners map variable acoustic-phonetic information onto their linguistic representations stored in the mental lexicon. The current study reports a mismatch negativity experiment, investigating the neural processing of Mandarin Chinese tone 3 sandhi words, to inform our understanding of the role of the acoustic input and the stored linguistic representations as listeners map the signal during spoken word recognition.

### Psycholinguistic Studies on Mandarin Chinese Tone 3 Sandhi

Mandarin Chinese (henceforth, Mandarin) is a tone language in which the fundamental frequency (F0) of syllables distinguishes word meanings. Mandarin has four tones. Tone 1 is a high-level tone (55); Tone 2 is a high-rising tone (35); Tone 3 is a low-dipping tone (214); and Tone 4 is a high-falling tone (51). In Mandarin, around 70 percent of words are disyllabic (Shei, 2014). For disyllabic compounds in Mandarin, words undergo a tonal alternation phenomenon called tone 3 sandhi in which an initial tone 3 syllable changes into a tone 2 when followed by another tone 3 syllable (Cheng, 1966, 1973; Chao, 1968; Dow, 1972; Shih, 1997; Peng, 2000; Duanmu, 2007; Lin, 2007). Acoustic analyses on the realization of this sandhi tone 3, compared to the canonical tone 2, has shown an overall lower F0 height (Peng, 2000), a lower F0 peak, as well as a later F0 rising onset (Yuan and Chen, 2014) for the sandhi tone 3, suggesting that the realization of sandhi tone 3 may be influenced by its underlying tone 3, and that sandhi tone 3 and canonical tone 2 are not completely neutralized in production. However, perception results have demonstrated that native speakers often cannot distinguish sandhi tone 3 from canonical tone 2 (Wang and Li, 1967; Peng, 2000), posing a mismatch between the production and perception of sandhi tone 3.

Zhou and Marslen-Wilson (1997) contrasted three alternative views concerning how tone sandhi words are represented in the mental lexicon. The canonical representation view states that tone sandhi words are represented as the concatenation of the citation forms of their constituent morphemes. Therefore, according to the canonical representation view, the Mandarin sandhi word [pɑw2 ɕjɛn3] 保險 “insurance”, would be represented as /pɑw3 ɕjɛn3/ in Mandarin speakers’ lexicon. The surface representation view posits that tone sandhi words are stored based on their surface form (represented as /pɑw2 ɕjɛn3/). The abstract representation view states that since the morphemes undergoing tone sandhi could have either of two surface tones, the lexical representations of them are underspecified, so that they are compatible and do not conflict with either alternation (for segmental underspecification, see Lahiri and Marslen-Wilson, 1991; Lahiri and Reetz, 2002, 2010). Zhou and Marslen-Wilson’s (1997) first auditory priming study on the processing of tone sandhi in Mandarin showed slower reaction times for tone 3 sandhi targets (e.g., [ts^h^aj2 tɕ^h^y3] /ts^h^aj3 tɕ^h^y3/ “take an action”) when preceded by disyllabic tone 2 primes (e.g., [ts^h^aj2 xua2] /ts^h^aj2 xua2/ “talent”) compared to when preceded by unrelated control primes (e.g., [t^h^jan1 ʰ2] /t^h^jan1 ʰ2/ “swan”), and faster reaction times when preceded by disyllabic tone 3 primes (e.g., [ts^h^aj3 xoη2] /ts^h^aj3 xoη2/ “rainbow”) compared to when preceded by the unrelated controls. Their second experiment showed slower reaction times for disyllabic targets with an initial tone 2 (e.g., [ts^h^aj2 p^h^an4] /ts^h^aj2 p^h^an4/ “referee”) when preceded by disyllabic tone 2 primes (e.g., [ts^h^aj2 tʂ^h^an3] /ts^h^aj2 tʂ^h^an3/ “asset”), tone 3 primes (e.g., [ts^h^aj3 na4] /ts^h^aj3 na4/ “adopt”), and sandhi primes (e.g., [ts^h^aj2 faη3] /ts^h^aj3 faη3/ “interview”) compared to when preceded by disyllabic unrelated control primes (e.g., [y4 ljao4] /y4 ljao4/ “predict”). In addition, the inhibitory effect was even stronger in the tone 3 prime condition. Zhou and Marslen-Wilson claimed that the results from these two experiments were not consistent with the abstract representation view, yet they could not distinguish between the canonical and surface representation views.

To further investigate how Mandarin tone 3 sandhi words are processed, Chien et al. (2016) conducted an auditory-auditory priming lexical decision experiment examining the role of surface and underlying forms. Each disyllabic Mandarin tone 3 sandhi target (e.g., /fu3 tao3/輔導, “to counsel”) was preceded by one of three corresponding monosyllabic primes, namely, a tone 2 prime (e.g., /fu2/服, “to assist”), a tone 3 prime (e.g., /fu3/輔, “to guide”), or a control prime (e.g., /fu1/敷, “to put on”). Results showed that tone 3 primes (underlying form overlap with the first syllable of the tone 3 sandhi targets) facilitated participants’ lexical decision responses, while tone 2 primes (surface form overlap with the first syllable of the tone 3 sandhi targets) did not show facilitation relative to the unrelated control primes. Moreover, these priming effects occurred regardless of the targets’ word frequency. These data show that tone 3 primes (e.g., /fu3/輔, “to guide”) facilitated reaction times to all tone sandhi targets (e.g., /fu3 tao3/輔導, “to counsel”) due to morpheme level activation between primes and the underlying form of the first syllable of the targets, while tone 2 primes (e.g., /fu2/服, “to assist”), although matching targets on the surface, did not show priming effects, indicating that surface matching did not result in faster sandhi word recognition. Chien et al. (2016) concluded that Mandarin tone 3 sandhi words are represented as /tone 3 tone 3/ in listeners’ mental lexicon.

Given that previous auditory priming studies haven’t yet reached consensus regarding how Mandarin tone 3 sandhi words are represented in the mental lexicon, the current study further investigated tone sandhi processing by examining cortical responses utilizing a mismatch paradigm, which has been argued to reflect pre-attentive processing of auditory linguistic representations independent of any behavioral task (Kujala et al., 2007; Näätänen et al., 2007). A number of previous studies have shown that the mismatch paradigm can be used to examine higher-level processes outside of the focus of attention (Phillips et al., 2000; Pulvermüller and Shtyrov, 2006; Gu et al., 2012). Additionally, using the mismatch paradigm also allowed us to isolate the effect elicited by the first tone of tone 3 sandhi words, so that we could examine how Mandarin listeners process the initial rising tone, which could potentially be the first syllable of a tone 3 sandhi word, during spoken word recognition.

### Neurophysiological Studies on Lexical Tone Processing

In the past few decades, event-related brain potentials (ERPs) have been used to investigate the neural basis of perception with high temporal resolution. Although ERPs do not transparently reflect where neural processes occur, they do provide a millisecond by millisecond record of brain activity, yielding a number of ERP components linked to perception. One ERP component that the speech perception literature has widely used in order to investigate the representation and processing of spoken language is the mismatch negativity (MMN). Previous studies have shown that the mismatch negativity (or mismatch field, MMF, in MEG) reflects deviations from previously presented events or regularities (Näätänen et al., 1997; Phillips et al., 2000; Kazanina et al., 2006; Kujala et al., 2007; Wang et al., 2012). MMN is traditionally recorded using an oddball paradigm in which a sequence of similar sounds (“standards”) is presented and rare sounds (“deviants”) are introduced occasionally. The similar sounds form a memory trace in the auditory system to which deviant sounds contrast, yielding MMN effects around 100–250 ms after the onset of deviant sound presentation (Näätänen, 1992; Näätänen and Winkler, 1999).

The mismatch response has also been used to examine the processing of tonal information in tone languages. Xi et al. (2010) used MMN to investigate categorical perception of Mandarin lexical tone 2 and tone 4 with the syllable structure /pa/. A 10-interval lexical tone continuum from tone 2 (stimulus 1) to tone 4 (stimulus 11) was created, producing a total of 11 stimuli. Based on the results of a previously conducted AX discrimination task, an across-category (stimulus 3 and stimulus 7) pair was implemented in an oddball experiment in which stimulus 3 was the deviant and stimulus 7 was the standard. In another oddball experiment, a within-category (stimulus 11 and stimulus 7) pair was selected, with stimulus 11 used as the deviant and stimulus 7 as the standard. Their results showed that two physically different tones across two distinct tonal categories elicited greater MMN amplitude than did two physically different tones within the same tonal category. Interestingly, physically different tones across tonal categories yielded a stronger MMN amplitude than did physically different tones within the same tonal category in the left hemisphere but not in the right hemisphere. These results suggest that the left-hemisphere may be more involved with the long-term phonemic processing of lexical tones while the right hemisphere may play a more important role for acoustic processing (Ren et al., 2009; Xi et al., 2010).

Research has also demonstrated that speakers can extract regularities from complex acoustic signals at the tonal level. Wang et al. (2012) conducted a mismatch negativity study to investigate whether Mandarin speakers possess a mechanism that can help them extract abstract linguistic rules from complex acoustic information at a pre-attentive stage. Their experiment was conducted using an oddball paradigm in which 90 percent of the monosyllabic stimuli were level tones (standards), carried by different Mandarin vowels ([i, e, a, u]), with various F0 height (10 levels from 78 Hz to 150 Hz) as well as intensity (3 intensity levels). The remaining 10 percent of the monosyllabic stimuli were either rising or falling tones (deviants), carried by Mandarin vowels ([i, e, a, u]), with three F0 levels (i.e., an identical F0 contour beginning at three different F0 heights) and intensity (same as the three intensities used for the standards). Results showed a mismatch negativity, indicating that the effect emerged when the rule formed by level tones was violated. Wang et al. (2012) concluded that listeners can extract linguistically meaningful rules from complex auditory signals during speech processing.

Recently, MMN studies have begun to examine representations involving phonological alternations. Li and Chen (2015) conducted a mismatch negativity study to examine Mandarin lexical tone representations involving phonological tonal alternations. An oddball experiment was conducted in which monosyllables [ma] with tone 1, tone 2 and tone 3 were set as stimuli. Four conditions were generated: T1 standard/T3 deviant, T3 standard/T1 deviant, T2 standard/T3 deviant, and T3 standard/T2 deviant. Results showed that all four conditions elicited MMN effects. However, the patterns of MMN effects were different. T1 standard/T3 deviant and T3 standard/T1 deviant yielded comparable MMN effects. However, T2 standard/T3 deviant and T3 standard/T2 deviant showed asymmetrical MMN effects. When T3 was the standard and T2 was the deviant, the MMN effect was significantly reduced compared to when T2 was the standard and T3 was the deviant. Moreover, the MMN elicited in the T3 standard/T2 deviant condition demonstrated a right hemispheric distribution, with all the other three conditions showing a left hemispheric MMN distribution. Li and Chen (2015) claimed that the reduced, right lateralized MMN effect in the T3 standard/T2 deviant condition may be due to the fact that when participants heard a tone 3 syllable, both tone 3 and tone 3V (the sandhi tone, which is acoustically similar to tone 2; Yuan and Chen, 2014) representations were activated, leading to the reduced MMN effect. Based on these results, Li and Chen (2015) proposed that tone 3 is represented as both /tone 3/ and /tone 3V/ in the mental lexicon.

Similar to Li and Chen (2015), Politzer-Ahles et al. (2016) recently investigated the nature of Mandarin tone 3 by looking at the MMN response to tone 3 in contrast with other tones. In three experiments, participants were presented with conditions where monosyllabic tone 3 words and monosyllabic words with other tones (tone 1, tone 2, tone 4) alternatively served as standards and deviants. Across the experiments, they found the predicted asymmetrical MMN response elicited by conditions with tone 3, while no asymmetry was observed in conditions with other tones. Mandarin tone 3 has several properties that underspecified segments also have, such as surface alternation, low pitch register, and use as attentional cues (Kuo et al., 2007; Hsu et al., 2015; Politzer-Ahles et al., 2016). Politzer-Ahles et al. (2016) argued that the putatively underspecified nature of tone 3 weakens the contrast between standards and deviants. Thus, when tone 3 contrasted with other tones, asymmetrical MMNs were observed. Politzer-Ahles and colleagues suggested that the findings are consistent with the underspecification accounts of Mandarin tone 3, in that listeners do not seem to assign a clear tonal category to tone 3 words. Although Li and Chen (2015) as well as Politzer-Ahles et al. (2016) provided evidence regarding Mandarin tone 3 representations, these patterns of data were observed with isolated syllables. A further question is how Mandarin native speakers process disyllabic tone sandhi words.

Zhang et al. (2015) began to address this question in a speech production task, using Mandarin disyllables to investigate the phonological encoding of Mandarin tone 3 sandhi words. Participants were asked to concatenate two auditorily presented syllables together and covertly pronounce them as a whole chunk (e.g., T2 + T3 and T3 + T3). Participants were then asked to overtly pronounce these disyllables, as a unit, after a silent interval (varying between 1000 and 1600 ms). Zhang et al.’s (2015) goal was to test whether Mandarin tone 3 sandhi words involve computational conversion of the first tone 3 syllable into a tone 2 syllable, or whether tone 3 sandhi words undergo a lexical mechanism in which a tone 3 sandhi word’s surface form is directly accessed.

ERP recordings acquired during covert production showed a significantly greater P200 (P2) amplitude at midline electrodes elicited by tone 3 syllables which are preceded by a tone 3 syllable than those preceded by a tone 2 syllable. The stronger P2 amplitude yielded by the second tone 3 syllable of Mandarin tone 3 sandhi words indicated that Mandarin speakers did expend additional effort, as P2 has been shown to be related to higher-order perceptual processing, including attention, repetition effects and task difficulties (Dunn et al., 1998; Kim et al., 2008; Landi et al., 2012). Moreover, no significant P2 difference was observed between real tone 3 sandhi words and tone 3 sandhi non-words, suggesting that Mandarin speakers process tone 3 sandhi words computationally, applying the tone 3 sandhi rule even to pseudowords.

However, the greater P2 effect elicited by the tone 3 syllables preceded by other tone 3 syllables could also be because it was easier for the participants to produce words (T2 + T3) that matched the two syllables they heard (T2 + T3) than to produce a word that did not match the two syllables they heard (T3 + T3). Since tone 3 syllables were always presented as a canonical falling-rising tone [214] rather than as their sandhi form [35], participants had to transform the canonical falling-rising tone 3 of the first syllable of a disyllabic sequence into a tone 2 in order to produce the correct output. Additionally, the fact that participants were provided the canonical tone 3 form might encourage them to focus on the underlying form (falling-rising F0 contour) of tone 3 in these sandhi syllables, which might then require participants to use extra effort to convert what they heard (a falling-rising F0 contour) to what they needed to produce (a rising F0 contour).

Taken together, previous neurophysiological studies on tone sandhi provide some differing evidence regarding the representation of Mandarin tones and how Mandarin tone 3 sandhi words are processed. While Li and Chen (2015) proposed that Mandarin tone 3 monosyllables should be represented as both tone 3 and tone 3V in the mental lexicon, Politzer-Ahles et al.’s (2016) data indicated that tone 3 may be phonologically underspecified, although neither study directly examined disyllabic tone 3 sandhi words.

The current study directly examines the processing of disyllabic tone sandhi words. In word recognition, Mandarin native speakers must process the tone of the first syllable of Mandarin tone 3 sandhi words, which is acoustically similar to a tone 2. Whether Mandarin speakers process the initial sandhi tone 3 of sandhi words as a tone 2 or a tone 3, or whether speakers assign a special status to the initial sandhi tone 3 and wait for the context (i.e., either a tone 3 syllable or not) is unclear. To address this issue, we conducted a mismatch negativity study to test the role of surface and underlying forms of Mandarin tone 3 sandhi words during spoken word processing. Investigating the processing of Mandarin tone 3 sandhi words in this way allows one to examine processing independent of any overt behavioral response, such as lexical decision.

### Current Study

**FIGURE 1:**
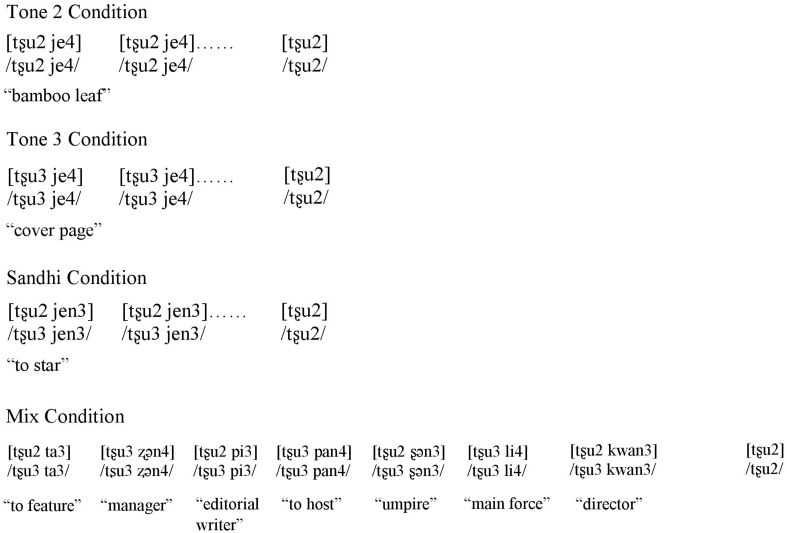
Stimuli in the Tone 2 condition, Tone 3 condition, Sandhi condition, and Mix condition.

An oddball paradigm was used to compare mismatch responses across four conditions: Tone 2, Tone 3, Sandhi, and Mix. In all conditions, the standards were disyllables and the deviant was a tone 2 monosyllable (see Figure 1). We utilized a varying-standard design in which the standards within each condition were not acoustically identical to each other, as our main interest in the current study is to examine the contexts in which the brain is able to extract a shared tonal representation across the physically different standards within each condition, rather than to test whether the brain can distinguish single tokens that either match or mismatch in tone.

The Tone 2 condition included disyllabic words with an initial tone 2 syllable followed by a tone 4 syllable ([tone 2 tone 4] /tone 2 tone 4/). The lack of a tone 3 sandhi environment in this condition ensured that the initial tone 2 of the standards had a surface and an underlying tone 2 form. In this condition, there is no mismatch between the standards and deviants in underlying tone category or surface tone category (see Figure 1), as both the initial syllable of standards and deviants are tone 2. However, as this study utilizes a design in which multiple physically different tokens of the standards are used, this condition is intended to assess whether the physical difference between the set of standards and deviants is sufficient to yield an MMN (Winkler et al., 1999; Kasai et al., 2001; Xi et al., 2010), or whether MMN will be absent due to the lack of many-to-one ratio at the level of tone category.

The Tone 3 condition included disyllabic standards with an initial tone 3 syllable followed by a tone 4 syllable ([tone 3 tone 4] /tone 3 tone 4/). Since none of the standards in this condition were sandhi words, the first syllable should be interpreted as tone 3 in both the surface and underlying form. For the Tone 3 condition, we expected to observe an MMN effect due to the mismatch between the first syllable of standards and the deviant in both underlying and surface forms (see Figure 1). Although previous studies using monosyllables had found a reduced MMN effect when a tone 3 was the standard and a tone 2 was the deviant (Li and Chen, 2015; Politzer-Ahles et al., 2016), we predicted that in the current study, the disyllabic tone 3 standards might reduce the possibility for Mandarin-speaking participants to activate a tone 3V or a phonologically underspecified representation because the initial tone 3 syllable is never followed by another tone 3 syllable (i.e., it is never in a sandhi context). The present disyllabic context would encourage participants to assign a clear tone 3 interpretation, potentially leading to a strong MMN effect in this condition.

The Sandhi condition included disyllabic tone 3 sandhi words ([tone 2 tone 3] /tone 3 tone 3/), with a tone 2 on the surface and a tone 3 underlyingly. For the Sandhi condition, whether or not an MMN effect would emerge depends on how participants process Mandarin tone 3 sandhi words. If participants process Mandarin tone 3 sandhi words based on the underlying tone 3 and the deviant is interpreted as a canonical tone 2 syllable in isolation, an MMN effect should be elicited since the tone 2 deviant would violate the regularity formed by repetitively occurring underlying tone 3 of the first syllable of standards. If participants focus on the surface tone 2, then no MMN effect would be expected due to the lack of tonal mismatch with the tone 2 deviant at the level of surface tone category (see Figure 1). If both the underlying tone 3 and the surface tone 2 are activated, then an MMN effect may still be observed, but with a weaker amplitude compared to that yielded in the Tone 3 condition.

In a fourth Mix condition, standards consisted of disyllabic tone 3 sandhi words ([tone 2 tone 3] /tone 3 tone 3/) mixed with disyllabic words starting with a tone 3 syllable ([tone 3 tone 4] /tone 3 tone 4/). In the Mix condition, whether or not an MMN effect would emerge depends on whether Mandarin-speaking participants could extract the underlying tone of the first syllable of standards despite the differences in surface tone. If they could, then an MMN effect is predicted because the tone 2 deviant would violate the regularity formed by frequently occurring underlying tone 3 of the first syllable of the standard, similar to Wang et al. (2012) where Mandarin-speaking participants extracted a level tone for standards and two contour tones for deviants despite their varying acoustic information, F0 heights, and intensity. If they could not extract the underlying tone of the first syllable of standards, then no MMN effect would be elicited (see Figure 1).

For all four conditions, the initial syllable of the standards was matched for segmental information, and the same tone 2 monosyllabic deviant, which also matched the segmental information of the first syllable of the standards, was used.

These four conditions allowed us to directly examine how Mandarin tone 3 sandhi words are processed by comparing the ERPs elicited by the deviant and those yielded by the first syllable of standards in the same condition. In addition, utilizing disyllabic standards in this MMN study allowed for explicitly testing the perception of tone 3 sandhi at a pre-attentive stage, and teasing apart the role of surface and underlying forms. Specifically, we were able to determine whether Mandarin speakers processed the first syllable of sandhi words as a tone 2 or a tone 3 by examining whether MMN is elicited by the deviant that contrasts with the first syllable of standards in each condition.

Regarding the three contrasting views on the representation of tone sandhi words (Zhou and Marslen-Wilson, 1997), the surface representation view would predict that the Sandhi condition and Tone 2 condition show similar MMN effects since the surface tonal information is matched across these two conditions, while the Sandhi condition compared to the Tone 3 condition and /or the Mix condition would yield different MMN patterns, given the different surface tonal information between the Tone 2 condition and these two conditions. The canonical or underlying representation view would predict similar MMN patterns for the Tone 3 condition, the Sandhi condition, and the Mix condition, since all these conditions possess an underlying tone 3 as the initial syllable of standards while the MMN pattern between the Sandhi condition and Tone 2 condition would be predicted to be different since these two conditions have different underlying tones as the initial syllable of standards. The abstract representation view would predict dissimilar MMN patterns among the Sandhi condition and all the other conditions given that it is the initial tone of the sandhi words that repeatedly alternates with tone 2, which would license an underspecified environment while the initial tone of standards in the other conditions does not consistently undergo tone sandhi.

Finally, the current design also allowed us to examine within-condition comparisons between the second syllable of standards and the omission position (following the monosyllabic deviant). Given that our standards were disyllabic and our deviants were monosyllables, we were able to utilize the omission MMN to directly investigate whether the deviants were processed as monosyllables or as the first syllable of an expected disyllabic word. In our study, we would thus expect to observe an omission MMN effect in all conditions given that the deviant is a monosyllable, which would violate the prediction formed by the disyllabic standards (Raij et al., 1997; Yabe et al., 1997, 1998; Janata, 2001). Additionally, we may also predict different omission MMN amplitudes across conditions given that the second syllable of standards used in the four conditions varies, which might lead to different predictability (Bendixen et al., 2009, 2012, 2014). Finding these omission effects would provide evidence that Mandarin-speaking participants were processing disyllabic standards as disyllabic stimuli and monosyllabic deviants as the first syllable of disyllabic stimuli.

## Materials and Methods

### Participants

Thirty-two right-handed native Mandarin speakers were recruited (14 males and 18 females), with ages from 18 to 35 years old. All participants were students from the University of Kansas with no reported language disability or hearing impairment. This research was reviewed and approved by the Human Subjects Committee at the University of Kansas. All participants were asked to provide informed consent before the EEG experiment and were paid 10 dollars per hour for their participation.

### Stimuli

Stimuli were produced by a male native Beijing Mandarin speaker and recorded in an anechoic chamber with a cardioid microphone (Electrovoice, model N/D767a) and a digital solid-state recorder (Marantz, model PMD671), using a sampling rate of 22,050 Hz at the University of Kansas. Stimuli used in this experiment were ten disyllabic Mandarin words and one monosyllabic Mandarin word. For the ten disyllabic words, one was a tone 2 + tone 4 word; five were tone 3 sandhi words, and four were tone 3 + tone 4 words. The monosyllable was a tone 2 word. All the disyllabic stimuli were normalized to 630 ms in duration (first syllable 280 ms + second syllable 350 ms), and the monosyllabic word was adjusted to 280 ms in duration. Intensity of all stimuli was normalized to 65 dB. All the manipulations were conducted using Praat (Boersma and Weenink, 2015).

### Design

Stimuli for each of the four test conditions were presented using the variable-standards structure in an oddball paradigm (see Figure 1). In each condition, 87.5 percent of the stimuli were standards, while 12.5 percent of the stimuli were deviants. In the Tone 2 condition, 7 physically distinct instances of [tʂu2 je4] /tʂu2 je4/ “bamboo leaf” were used as standards. In the Tone 3 condition, 7 physically different tokens of [tʂu3 je4] /tʂu3 je4/ “cover page” were selected as standards. In the Sandhi condition, 7 physically different instances of [tʂu2 jen3] /tʂu3 jen3/ “to star” were set as standards. In the Mix condition, four tone 3 sandhi words (T3 + T3 tonal sequence) ([tʂu2 ta3] /tʂu3 ta3/ “to feature”; [tʂu2 pi3] /tʂu3 pi3/ “editorial writer”; [tʂu2 ʂɕn3] /tʂu3 ʂɕn3/ “chief umpire”; [tʂu2 kwan3] /tʂu3 kwan3/ “director”) and three disyllabic words with a T3 + T4 tonal sequence ([tʂu3 ʐɕn4] /tʂu3 ʐɕn4/ “manager”; [tʂu3 pan4] /tʂu3 pan4/ “to host”; [tʂu3 li4] /tʂu3 li4/ “main force”) were used as standards. For all four conditions, monosyllable [tʂu2] /tʂu2/ “bamboo” was used as the deviant.

Critically, in the Tone 2 condition, the first syllable of the standard [tʂu2 je4] /tʂu2 je4/ and the deviant [tʂu2] /tʂu2/ were matched in both the surface and underlying forms, as shown in Figure 1. In the Tone 3 condition, the first syllable of the standard [tʂu3 je4] /tʂu3 je4/ did not match the tone in the deviant [tʂu2] /tʂu2/ in either surface or underlying form. In the Sandhi condition, the first syllable of the standard [tʂu2 jen3] /tʂu3 jen3/ shared only the surface form with the deviant [tʂu2] /tʂu2/ but mismatched in the underlying form (tone 3). In the Mix condition, there was not a many-to-one ratio with respect to the first syllable of the standards and the deviant on the surface (either tone 2 or tone 3). However, there was a many-to-one ratio between the first syllable of the standards and the deviant underlyingly (all tone 3).

To equate the Tone 2 and Sandhi conditions, tonal contours from four canonical tone 2 (four of the seven first syllables of the [tʂu2 je4] /tʂu2 je4/ standards) in the Tone 2 condition were superimposed upon the first syllable of 4 standards ([tʂu2 jen3] /tʂu3 jen3/) in the Sandhi condition. Likewise, tonal contours from three sandhi tones (three of the seven first syllables of the [tʂu2 jen3] /tʂu3 jen3/ standards) in the Sandhi condition were superimposed on the first syllable of the other 3 standards ([tʂu2 je4] /tʂu2 je4/) in the Tone 2 condition. Therefore, the first syllable of standards in the Tone 2 and Sandhi conditions had identical tonal contours, with some from underlying tone 2 stimuli and some from tone 3 sandhi stimuli.

For the Tone 3 condition, tonal contours of the standards were not manipulated. In the Mix condition, two of the four standards were the same as the first syllable of the two [tʂu2 je4] /tʂu2 je4/ Tone 2 condition standards; two of the four were the same as the first syllable of the two [tʂu2 jen3] /tʂu3 jen3/ Sandhi condition standards. By doing so, the first syllable of standards in the Mix condition had similar tone 2 contours as the first syllable of standards in the Tone 2 and Sandhi conditions. For the other three T3 + T4 words, no manipulation was conducted. The second syllables were not manipulated either, with four tone 3 syllables and three tone 4 syllables varying in terms of the segmental information.

The same monosyllabic [tʂu2] was used as the deviant across the four conditions. The tonal contour of the deviant was not manipulated.

Fundamental frequency (F0) was measured for the seven standard [tʂu2] tokens and the deviant [tʂu2] in the Tone 2 and Sandhi conditions. The fundamental frequency of the seven standard [tʂu2] tokens in the Tone 2 and Sandhi conditions was, on average, 10.28 Hz higher than that of the deviant [tʂu2] in these two conditions. The ΔF0 value (i.e., the change in fundamental frequency from onset of the tone to the lowest pitch value) of the average F0 contour of the standard [tʂu2] tokens was 9.93 Hz, while the ΔF0 value of the deviant [tʂu2] was 15.23 Hz. Similar measures were also collected for the seven standard [tʂu3] tokens in the Tone 3 condition. The F0 of the [tʂu3] tokens was realized as a low-falling tone, which was distinct from that of the deviant [tʂu2]. Acoustic details of the stimuli are shown in Appendix A.

The four conditions were separately presented in 8 blocks, with each condition being split into two blocks. The order of the four conditions was counterbalanced across participants. At the beginning of each block, 14 standards were presented first to familiarize the participants with the standards. Then another 400 trials were presented in a pseudo-randomized order, with every two deviants separated by 2 to 9 standards. There was a 600 ms pause between stimuli. In total, participants heard 828 stimuli in each condition, with 728 standards and 100 deviants. A self-paced break was offered after each block.

### Procedure

Participants were invited to the Neurolinguistics & Language Processing Laboratory at The University of Kansas. They filled out a consent form, the Edinburgh Handedness Inventory (Oldfield, 1971), as well as a language background questionnaire. The participants were then fitted with an electrode cap before they were seated in front of a computer screen. Auditory stimuli were presented using insert earphones (Etymotic Research, Inc.) while participants watched a silent movie with Chinese subtitles on the computer screen in front of them (Chandrasekaran et al., 2007, 2009; Xi et al., 2010; Gu et al., 2012; Wang et al., 2012; Politzer-Ahles et al., 2016). Participants were instructed to ignore the auditory stimuli while watching the movie. The total duration of the experiment was around 2 h.

### EEG Recordings

Electroencephalogram (EEG) was recorded at a sampling rate of 1 kHz using an electrode cap (Electro-cap International, Inc.) from 32 sintered, Ag/AgCl scalp electrodes, arranged in a modified 10–20 layout. Electrode sites were FPz, Fz, FCz, Cz, CPz, Pz, Oz, FP1/2, F7/8, F3/4, FT7/8, FC3/4, T3/4, C3/4, TP7/8, CP3/4, T5/6, P3/4, and O1/2, with electrode AFz serving as the ground electrode and the left mastoid as online reference. The recording was amplified by using a Neuroscan Synamps2 amplifier system (Compumedics Neuroscan, Inc.). Additional electrodes were placed above and below each eye, and on the left and right outer canthi of each eye to detect blinks and eye movements. Impedances were kept below 5 kOhms. Data were continuously recorded in AC mode using an online high-pass filter of 0.1 Hz and a low-pass filter of 200 Hz.

### Data Analysis

Event-related brain potentials analyses were time-locked at the onset of the first syllable of standards (0 ms post onset of standard presentation), the onset of deviants (0 ms post onset of deviant presentation), the onset of the second syllable of standards (280 ms post onset of standard presentation), and the onset of omission positions (280 ms post onset of deviant presentation). Continuous EEG data were re-referenced offline to the mean of the left and right mastoids using Neuroscan Edit (Compumedics Neuroscan, Inc.). The subsequent analyses were carried out using EEGLAB (Delorme and Makeig, 2004) and MATLAB (The MathWorks, Inc.). Bad channels were interpolated; no more than 2 bad channels were found per participant. The continuous data were then segmented into −500 ms to 500 ms epochs relative to all the triggers and demeaned using the mean of the whole epoch (as recommended by Groppe et al., 2009). The data were then decomposed into independent components using ICA (Makeig et al., 1996). For each participant one to five independent components that are typical for eye movements, blinks, or muscle artifacts were identified by visual inspection and pruned from the data.

Trials were then divided into smaller epochs based on Status (standard, deviant), Condition (Tone 2, Tone 3, Sandhi, Mix) and Position (first syllable/onset, second syllable/omission) resulting in 16 different combinations. The epoch for the first syllable of standards and deviants at the onset position in all four conditions spanned a −100 ms to 400 ms time window with respect to the onset of the first syllable (0 ms). The epoch for the second syllable of standards and deviants at the omission position in all four conditions spanned a −380 ms to 400 ms time window with respect to the onset of the second syllable (0 ms). The first-syllable-of-standard epochs and deviant epochs were then baseline-corrected using the −100 ms to 0 ms interval preceding the first syllable, while the second-syllable-of-standard epochs and omission epochs were baseline-corrected using the −380 ms to −280 ms interval (this part of the epoch was used for baseline correction as it is the 100-ms interval preceding the first syllable). Epochs with amplitudes exceeding ± 100 μV at any channel were automatically rejected, resulting in the elimination of two participants’ data due to excessive artifacts (i.e., more than 20% of deviant trials at both onset and omission positions were excluded). For the remaining 30 participants, less than 2% of all the trials were excluded. Epochs were then low-pass filtered at 30 Hz. The remaining data were carried forward for statistical analysis. Averaged ERP waveforms were generated for the first-syllable-of-standard epochs, deviant epochs, second-syllable-of-standard epochs, and omission epochs within each condition.

The traditional MMN method was employed by comparing the ERPs elicited by the deviant with those by the standards in the same condition, since the main focus of this study is to compare responses to the same tone 2 deviant following different standard contexts, rather than having the same tone 2 stimuli as standards and deviants in different conditions. Moreover, note that the monosyllabic deviant [tʂu2] would be ambiguous if it was treated as the standard in a reverse block used for the same-stimulus MMN method (i.e., comparing the ERPs to the deviant with those to the same stimulus when it is presented as the standard in another block). Such ambiguity was unlikely to happen when the surface form [tʂu2] served as the deviant in the current study, since the participants would interpret the deviant [tʂu2] based on their predictions of the upcoming stimuli, and these predictions were made based on the standard of each condition. The same-stimulus method would not allow us to compare the same status of [tʂu2] within each condition. Moreover, previous research showed that the traditional and same-stimulus methods produced similar MMN patterns yielded by frequency (Peter et al., 2010), and we also compared responses to the physically identical deviant [tʂu2] across conditions to ensure that the results were compatible with those generated by comparing the MMN effects across conditions using the traditional method.

The midline electrode Fz was selected for mean ERP amplitude analyses since previous studies have shown that mismatch effects have a fronto-central distribution (Näätänen et al., 2007; Ren et al., 2009; Gu et al., 2012). Additionally, we analyzed the mean ERP amplitudes on 9 representative electrodes (F3, Fz, F4, C3, Cz, C4, P3, Pz, and P4) in order to characterize the topographic distribution of the MMN effects we may observe. For each participant, mean ERP amplitude was calculated by averaging the ERP voltages in the 100–280 ms time window [comparable to that used in Li and Chen (2015) and Politzer-Ahles et al. (2016)] across epochs by Status (standard vs. deviant), Condition (Tone 2, Tone 3, Sandhi, and Mix) and Position (onset vs. omission).

## Results

Statistical analyses were conducted on the mean ERP amplitudes obtained from the MMN experiment. The MMN results generated by comparing the mean ERP amplitudes at the deviant position and those at the first syllable of standards will be presented first. Then the MMN results produced by comparing the mean ERP amplitudes at the omission position and those at the second syllable of standards will be introduced.

### Deviant Onset Position

#### Fz Analysis

Mean ERP amplitudes were analyzed via a series of linear mixed-effects models using the lme4 package (Bates et al., 2015) in R, with *p*-values calculated by the *lmerTest* package (Kuznetsova et al., 2017). Status (First Syllable of Standard, Deviant) and Condition (Tone 2, Tone 3, Sandhi, Mix) were entered as categorical fixed factors. For Status, Deviant was set as the baseline to which First Syllable of Standard was compared in order to derive MMN, while for Condition, Tone 2 was coded as the baseline. Participant was included as the random intercept, while Status and Condition were entered for by-Participant random slopes. A series of likelihood ratio tests were performed to compare three by-Participant random slopes models in order to evaluate effects of Status, Condition, and Status × Condition. Results showed that the model containing Status, Condition, as well as Status × Condition was significantly better than the one consisting of Status and Condition (χ*^2^* = 24.311, *df* = 3, *p* < 0.001), thus, determined to be the best model. Given that Status (First Syllable of Standard, Deviant) is the crucial variable to index MMN effects, only the results relevant to Status are reported below.

**FIGURE 2:**
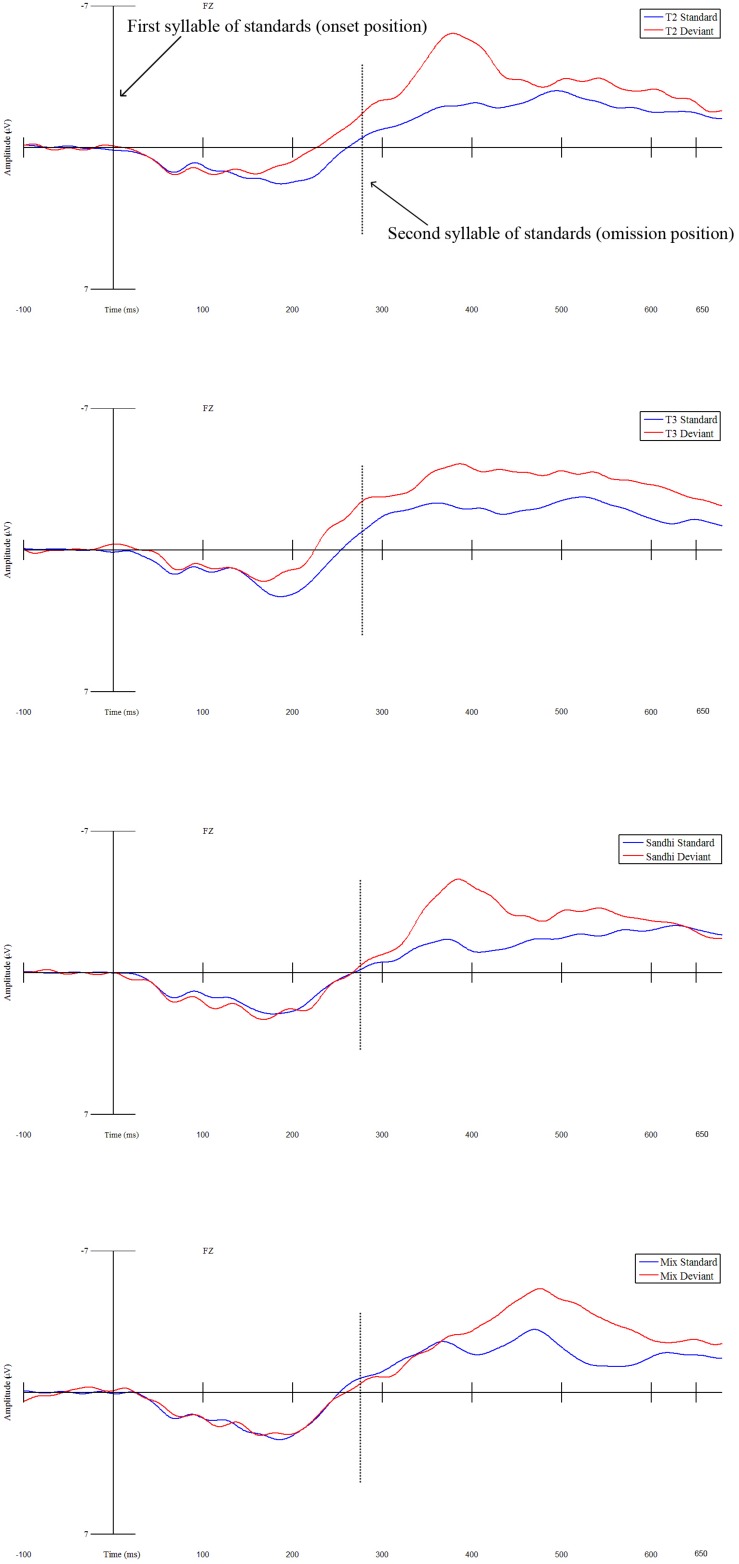
The four panels depict mean ERP waveforms for electrode Fz averaged across 30 participants in the 0–680 ms time window (using the –100–0 ms time window as the baseline) for the four experimental MMN conditions (Tone 2 condition is the top panel, Tone 3 condition is the second panel, Sandhi condition is the third panel, Mix condition is the bottom panel). The solid vertical line delineates the onset of the first syllable of the standards (blue line) and the onset of the deviants (red line). The dotted line delineates the end of the first syllable of standards which is also the onset of the second syllable of standards (blue line) and the offset of the deviants which is also the onset of omissions (red line).

**FIGURE 3:**
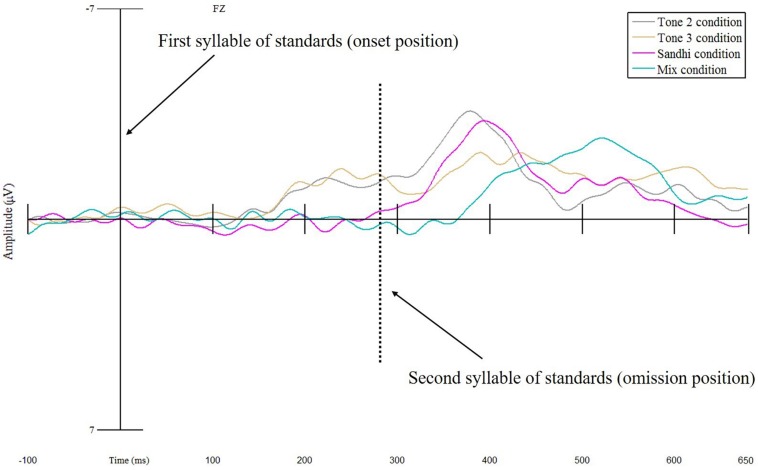
Mean MMN difference waves (Deviant – Standard) for electrode Fz for the Tone 2 condition, Tone 3 Condition, Sandhi condition, and Mix condition at both onset and omission positions.

As for the best model, linear-mixed effects analysis demonstrated that ERPs elicited by the deviant were significantly more negative than those produced by the first syllable of standards, indicating an MMN effect (β = 0.744, *SE* = 0.187, *t* = 3.987, *p* < 0.001) (see Figure 2). Moreover, a significant interaction between Status and Condition was also obtained (*df* = 3, *F* = 8.696, *p* < 0.001). This interaction effect resulted from the significantly stronger MMN effect in the Tone 2 condition than that in the Sandhi condition (β = −0.903, *SE* = 0.255, *t* = −3.543, *p* < 0.001), and than that in the Mix condition (β = −0.812, *SE* = 0.255, *t* = −3.1888, *p* = 0.002). However, the MMN effect in the Tone 2 condition was not different from the MMN effect in the Tone 3 condition (β = 0.114, *SE* = 0.255, *t* = 0.447, *p* = 0.656) (see Figure 3).

Given that we observed a main effect of Status with all conditions included, we conducted four follow-up linear-mixed effects analyses between First Syllable of Standard and Deviant to evaluate whether MMN effects emerged in each individual condition (see Figure 2). For each analysis, mean ERP amplitude was regarded as a dependent variable, while Status (First Syllable of Standard, Deviant) was treated as a categorical fixed factor, with Deviant coded as the baseline in order to derive MMN. In addition, Participant was set as a random intercept, while Status was coded for by-participant random slopes.

Results showed that for Tone 2 condition, ERPs elicited by the deviants were significantly more negative than those yielded by the first syllable of standards (β = 0.744, *SE* = 0.226, *t* = 3.290, *p* = 0.003), indicating an MMN effect. For Tone 3 condition, ERPs yielded by the deviants were significantly more negative than those produced by the first syllable of standards (β = 0.858, *SE* = 0.153, *t* = 5.591, *p* < 0.001), also confirming an MMN effect. However, with regard to Sandhi condition, ERPs elicited by the deviants were not significantly different from those yielded by the first syllable of standards (β = −0.159, *SE* = 0.192, *t* = −0.829, *p* = 0.414), nor were ERPs yielded by the deviants in the Mix condition compared to those produced by the first syllable of standards in the Mix condition (β = −0.068, *SE* = 0.190, *t* = −0.360, *p* = 0.721) (see Figure 2).

Since the deviant was physically identical across all the four conditions, we could directly compare ERPs elicited by the deviant across conditions. We ran an additional mixed-effects analysis on the mean ERP amplitude of the deviant for Fz, with Condition as a fixed factor (i.e., Tone 2 condition was set as the baseline condition) and Participant as a random effect. The results patterned the same as those reported above (see Figure 3), with the mean ERP amplitude of the deviant in the Tone 2 condition being significantly more negative than that in the Sandhi (β = 1.014, *SE* = 0.250, *t* = 4.065, *p* < 0.001) and Mix conditions (β = 0.954, *SE* = 0.250, *t* = 3.823, *p* < 0.001).

#### Multi-Electrode Analysis

For the multi-electrode analyses, 9 representative electrodes were selected: F3, Fz, F4, C3, Cz, C4, P3, Pz, and P4. The electrodes were coded for Laterality (Left, Midline, Right) and Anteriority (Frontal, Central, Posterior): F3 as Left Frontal, Fz as Midline Frontal, F4 as Right Frontal, C3 as Left Central, Cz as Midline Central, C4 as Right Central, P3 as Left Posterior, Pz as Midline Posterior, and P4 as Right Posterior. A baseline level in each of these two variables was chosen to which the other levels were compared: Laterality: Left; Anteriority: Posterior. Mean ERP amplitudes were analyzed via linear mixed-effects models using the *lme4* package (Bates et al., 2015) in R, with *p*-values calculated by the *lmerTest* package (Kuznetsova et al., 2017).

A series of linear-mixed effects analyses were conducted. The dependent variable in the model was the mean ERP amplitude in the 100–280 ms time window at a given electrode. Likelihood ratio tests were conducted to evaluate these fixed effects: Status (First Syllable of Standard vs. Deviant in order to derive MMN), Condition (Tone 2, Tone 3, Sandhi, Mix), Laterality (Left, Midline, Right), Anteriority (Frontal, Central, Posterior), Status × Condition, Status × Anteriority, Status × Laterality, Condition × Anteriority, Condition × Laterality, Anteriority × Laterality, Status × Condition × Anteriority, Status × Condition × Laterality, Status × Anteriority × Laterality, Condition × Anteriority × Laterality, and Status × Condition × Anteriority × Laterality. Fifteen models were created by adding one of these fixed variables at a time and were compared via likelihood ratio tests; the model which included the most variables and fit significantly better than the one with one less variable was chosen as the optimal model. Participant was included as the random intercept, while Status and Condition were entered for by-Participant random slopes in all the models. Results showed that the model containing Status, Condition, Anteriority, Laterality, Status × Condition, Status × Anteriority, Status × Laterality, and Condition × Anteriority was the best fit (χ*^2^* = 16.582, *df* = 6, *p* = 0.011). The other more complex models failed to explain more variance of the ERP data. Given that Status is the crucial variable to index MMN effects, only the results relevant to Status are reported below.

Within the best model, a main effect of Status indicated that ERPs elicited by the deviant were significantly more negative than those yielded by the first syllable of standards, confirming the presence of an MMN effect overall (β = 0.561, *SE* = 0.113, *t* = 4.980, *p* < 0.001). A Status × Condition interaction suggested that the MMN effect was significantly stronger in the Tone 2 condition than in the Sandhi (β = −0.745, *SE* = 0.080, *t* = −9.277, *p* < 0.001) and Mix conditions (β = −0.743, *SE* = 0.080, *t* = −9.246, *p* < 0.001). However, the MMN effect in the Tone 2 condition did not differ from that in the Tone 3 condition (β = 0.037, *SE* = 0.080, *t* = 0.465, *p* = 0.642). We also found that the MMN effect yielded by the frontal electrode was significantly more negative than that by the posterior electrode (β = 0.145, *SE* = 0.070, *t* = 2.087, *p* = 0.037), indicating a frontally distributed MMN effect.

To summarize, both Fz and multi-electrode analyses showed that MMN effects were observed in the 100–280 ms time window for the Tone 2 and Tone 3 conditions, indicating that Mandarin speaking participants treated the deviants in the Tone 2 and Tone 3 conditions differently from the first syllable of standards in these two conditions. The MMN effect observed was frontally distributed. However, no MMN effect was obtained in the 100–280 ms time window for the Sandhi or Mix condition. In these two conditions, Mandarin-speaking participants seem to process the deviant and the first syllable of standards similarly.

### Deviant Omission Position

#### Fz Analysis

Similar to the onset position analyses, a series of linear-mixed effects analyses were conducted on the mean ERP amplitudes elicited by the omission position and the second syllable of standards within a 100–280 ms time window using the lme4 package in R (Bates et al., 2015), with *p*-values calculated by the *lmerTest* package (Kuznetsova et al., 2017). Status (Second Syllable of Standard, Omission) and Condition (Tone 2, Tone 3, Sandhi, Mix) were set as categorical fixed factors. For Status, Omission was treated as the baseline to which Second Syllable of Standard was compared in order to derive omission MMN. For Condition, Tone 2 was entered as the baseline. Participant was coded as random intercepts, while Status and Condition were entered as by-Participant random slopes. Likelihood ratio tests were conducted to compare among a series of linear-mixed effects models in order to evaluate effects of Status, Condition, as well as the Status × Condition interaction. Three models were created by adding one of these fixed variables at a time and were compared between each other; the model which included the most variables and fit significantly better than the one with one less variable was chosen as the optimal model. Results showed that the best model contained Status and Condition (χ*^2^* = 14.854, *df* = 3, *p* = 0.002), suggesting that the model consisting of Status, Condition, and Status × Condition interaction failed to explain more variance of the ERP data than the best model (χ*^2^* = 1.766, *df* = 3, *p* = 0.622). Such a result demonstrated that mean MMN amplitudes in the 100–280 ms time window did not statistically differ across conditions (see Figure 3). Below, only the effects related to Status will be presented.

With respect to the best model, there was an effect of Status, indicating that ERPs elicited by the omission position were significantly more negative than those yielded by the second syllable of standards (β = 1.706, *SE* = 0.237, *t* = 7.186, *p* < 0.001). Four follow-up linear mixed-effects analyses were performed to further examine whether MMN effects emerged in each condition by comparing mean ERP amplitudes related to the omission position with those related to the second syllable of standards within each condition. Moreover, Participant was set as a random intercept, while Status (Second Syllable of Standard, Omission) was entered for by-participant random slopes. Results showed that the ERPs yielded by the omission position were significantly more negative than those elicited by the second syllable of standards in all the four conditions, confirming MMN effects (Tone 2 condition: β = 1.459, *SE* = 0.348, *t* = 4.187, *p* < 0.001; Tone 3 condition: β = 1.688, *SE* = 0.358, *t* = 4.722, *p* < 0.001; Sandhi condition: β = 1.753, *SE* = 0.277, *t* = 6.325, *p* < 0.001; β = 1.925, *SE* = 0.352, *t* = 5.463, *p* < 0.001) (see Figure 2).

#### Multi-Electrode Analysis

Similar to the onset analyses, the omission multi-electrode analyses used 9 representative electrodes: F3, Fz, F4, C3, Cz, C4, P3, Pz, and P4. A series of linear-mixed effects analyses were conducted on mean ERP amplitudes at the 100–280 ms time window of a given electrode using the *lme4* package (Bates et al., 2015) in R, with *p*-values calculated by the *lmerTest* package (Kuznetsova et al., 2017). Status (Second Syllable of Standard vs Omission in order to derive omission MMN), Condition (Tone 2, Tone 3, Sandhi, Mix), Anteriority (Frontal, Central, Posterior) and Laterality (Left, Middle, Right) were entered as categorical fixed factors in which Omission, Tone 2, Posterior and Left were selected as the baseline for each factor. Participant was included as the random intercept, while Status and Condition were used as by-Participant random slopes. Likelihood ratio tests were performed to evaluate effects of Status, Condition, Anteriority, Laterality, Status × Condition, Status × Anteriority, Status × Laterality, Condition × Anteriority, Condition × Laterality, Anteriority × Laterality, Status × Condition × Anteriority, Status × Condition × Laterality, Status × Anteriority × Laterality, Condition × Anteriority × Laterality, and Status × Condition × Anteriority × Laterality. Fifteen models were created by adding one of these fixed variables at a time and were compared via likelihood ratio tests; the model which included the most variables and fit significantly better than the one with one less variable was chosen as the optimal model. Results showed that the model comprising Status, Condition, Anteriority, Laterality, Status × Condition, Status × Anteriority, Status × Laterality, Condition × Anteriority was the best (χ*^2^* = 23.838, *df* = 6, *p* < 0.001). The other more complicated models could not explain more variance of the ERP data. Given that Status, which indexes MMN effects, is most relevant to the current research interests, only those effects involving Status will be presented.

Within the best model, an effect of Status was obtained, as evidenced by the fact that ERPs elicited by the omission position were significantly more negative than those yielded by the second syllable of standards (β = 0.534, *SE* = 0.214, *t* = 2.497, *p* = 0.016). Furthermore, a Status × Condition interaction was also obtained, as revealed by significantly stronger omission MMN effects for the Tone 2 condition than for the Tone 3 condition (β = 0.265, *SE* = 0.098, *t* = 2.695, *p* = 0.007), the Sandhi condition (β = 0.229, *SE* = 0.098, *t* = 2.328, *p* = 0.020), and the Mix condition (β = 0.382, *SE* = 0.098, *t* = 3.888, *p* < 0.001). There was also an interaction between Status and Anteriority, showing significantly stronger omission MMN effects for frontal and central electrodes than for posterior electrodes (Frontal vs Posterior: β = 0.866, *SE* = 0.085, *t* = 10.166, *p* < 0.001; Central vs. Posterior: β = 0.493, *SE* = 0.085, *t* = 5.787, *p* < 0.001), suggesting a more frontal distribution.

In summary, the results revealed that omission MMN effects were elicited by the omission position in all four conditions, and were frontally distributed. Moreover, the omission effect in the Tone 2 condition was significantly greater than that in the Tone 3 condition, Sandhi condition, and Mix condition.

## Discussion

### Onset MMN

The current study used the mismatch negativity to examine the processing of phonological alternation. Specifically, we investigated how Mandarin speakers process and represent Mandarin tone 3 sandhi words using MMN. At the deviant onset position, we observed frontally distributed MMN effects only in the Tone 2 and Tone 3 conditions, but not in the Sandhi or Mix condition. Moreover, the MMN effects in the Tone 2 and Tone 3 conditions did not differ. These results were consistent with the analysis that compared the ERPs elicited by the deviant across the four conditions. In particular, we found that the ERP yielded in the Tone 2 condition did not differ from that in the Tone 3 condition, but was significantly more negative in amplitude than that in the Sandhi and Mix conditions.

Most importantly for understanding the processing of sandhi words, we found a significant difference between the Tone 2 condition (standard [tʂu2 je4] /tʂu2 je4/ and deviant [tʂu2] /tʂu2/) and the Sandhi condition (standard [tʂu2 jen3] /tʂu3 jen3/ and deviant [tʂu2] /tʂu2/) in that an MMN effect emerged in the Tone 2 condition but not in the Sandhi condition. Recall that the acoustic information of the first syllable of standards in these two conditions was identical. Moreover, the deviants used in these two conditions were also identical. Despite this manipulation, we only observed an MMN effect in the Tone 2 condition but not in the Sandhi condition. This pattern of data suggests distinct neural mechanisms when processing tone 2 standards ([tʂu2 je4] /tʂu2 je4/) and sandhi standards ([tʂu2 jen3] /tʂu3 jen3/).

The lack of an MMN effect in the Sandhi condition may be due to the representation of Mandarin tone 3 sandhi. Recall that Mandarin tone 3 sandhi is triggered by the phonological environment, that is, a tone 3 syllable undergoes tone sandhi, changing into a tone 2 syllable when followed by another tone 3 syllable. For our Sandhi condition, participants heard many tone 3 sandhi words ([tʂu2 jen3] /tʂu3 jen3/). Since no MMN effect was observed when hearing a tone 2 deviant in the Sandhi condition, participants did not seem to treat the standards as either a tone 2 (tone 3V) or a tone 3; otherwise, an MMN effect should have been elicited as in the Tone 2 and Tone 3 conditions. This result pattern contrasts to Li and Chen (2015) in which both sandhi tone 3V and tone 3 were reported to be activated. For the Sandhi condition, participants seem to be assigning an underspecified representation for the first syllable of the tone 3 sandhi words, which is neither a tone 2 nor a tone 3. The deviant ([tʂu2] /tʂu2/) did not elicit an MMN effect in the Sandhi condition even though the same acoustic differences between the first syllable of sandhi standards and the deviant yielded such an effect in the Tone 2 condition. This pattern is consistent with a proposal that Mandarin tone 3 may be underspecified. ERP studies (Politzer-Ahles et al., 2016) also offered evidence for the underspecified nature of tone 3, showing that monosyllabic tone 3 words, as contrasted with words with other tones, elicited asymmetrical MMN effects in an oddball paradigm. However, as Politzer-Ahles et al. (2016) also note, unlike coronal place of articulation which has several underspecified properties (Lahiri and Reetz, 2002, 2010), the falling-rising F0 contour of Mandarin tone 3 is one of the most extreme tones to produce, the most difficult tone to acquire for children (Wong et al., 2005; Wong, 2012), and the most infrequent tone in token frequency (Zhang and Lai, 2010), suggesting that more research is necessary to explore what acoustic properties of Mandarin tone 3 might contribute to its underspecified representation.

Another possible explanation for the lack of MMN effects in the Sandhi condition may be that participants productively applied the tone 3 sandhi rule by always converting the initial surface tone 2 into an underlying tone without exception (even for the deviant [tʂu2] /tʂu2/). Given that participants heard the same sandhi word many times in this condition, they might have always predicted the upcoming stimulus to be a sandhi word, thus interpreting even the deviant [tʂu2] /tʂu2/ as the first syllable of the sandhi standard, focusing mainly on the underlying tone. Therefore, when hearing the deviant [tʂu2], participants may have ignored the surface form and extracted its underlying tone, so that it would not be in conflict with the first syllable of sandhi standards, thus resulting in no MMN effect. Future research about the nature of the abstract representation of tone sandhi (as either underspecified or as a tone 3) is needed to differentiate these two accounts. If the lack of an MMN effect in the Sandhi condition was really due to the above-mentioned reason, it would be consistent with Chien et al.’s (2016) priming results in that surface tonal overlap between primes and targets did not significantly facilitate Mandarin-speaking participants’ lexical decision responses while underlying tonal overlap between primes and targets did show significant facilitation. It would further suggest that models of spoken word recognition that rely solely on surface forms need to be modified.

For the Tone 2 condition, notice that no sandhi environment existed in this condition. It is unlikely for the Mandarin listeners to posit that the tone 2 deviant was derived via the sandhi process, so that the first syllable of the standard [tʂu2 je4] /tʂu2 je4/ and the deviant [tʂu2] /tʂu2/ should match in both surface and underlying forms. However, our results showed an MMN effect in the Tone 2 condition. The significant MMN was most likely due to the acoustic differences between the deviants and the first syllable of standards. Recall that the average fundamental frequency of the seven [tʂu2] tokens of the standards in the Tone 2 condition was 10.28 Hz higher than that of the deviant [tʂu2]. In addition, the ΔF0 value of the average F0 contour of the standard [tʂu2] was 9.93 Hz, while the ΔF0 value of the deviant [tʂu2] was 15.23 Hz. The significant MMN effect in the Tone 2 condition is consistent with previous studies (with tones and with vowels) showing MMN effects when deviants and standards differed acoustically (within the same phoneme category) but not phonemically (in different phoneme categories) (Winkler et al., 1999; Kasai et al., 2001; Xi et al., 2010).

With respect to the Tone 3 condition, there was no sandhi context either. It is unlikely for the Mandarin listeners to expect that the tone 2 deviant came from the sandhi process. Therefore, the mismatch between the first syllable of tone 3 standards ([tone 3 tone 4] /tone 3 tone 4/) and the deviant ([tʂu2] /tʂu2/) in both surface and underlying forms yielded a significant MMN effect. Being regularly exposed to the standards [tʂu3 je4] /tʂu3 je4/ in the Tone 3 condition, Mandarin-speaking participants interpreted the first syllable they heard in a trial to be a tone 3. Encountering the deviant [tʂu2] /tʂu2/, mismatching both on the surface and underlyingly, resulted in a significantly more negative ERP waveform relative to that yielded by the first syllable of the tone 3 standards ([tʂu3 je4] /tʂu3 je4/).

However, the present data show no difference in MMN amplitudes between the Tone 2 and Tone 3 conditions. Previous neurophysiological studies have demonstrated that across-category differences elicited stronger mismatch responses than within-category differences (Dehaene-Lambertz, 1997; Winkler et al., 1999; Sharma and Dorman, 2000; Kasai et al., 2001; Nenonen et al., 2003; Xi et al., 2010). The pattern of results could be due to the acoustic information of the tone 2 deviant, the first syllable of the T2T4 standards, and the first syllable of the T3T4 standards. Notice that there were acoustic differences between the first syllable of T2T4 standards and tone 2 deviant in average F0 and ΔF0. Moreover, a lower average F0 and a larger ΔF0 value are the acoustic characteristics of tone 3, and the tone 2 deviant happened to have these acoustic characteristics. The fact that the tone 2 deviant was acoustically different from the first syllable of the T2T4 standards and had some acoustic traits similar to tone 3 may be able to explain why there was no MMN difference between the Tone 2 and Tone 3 conditions. An alternative explanation for the lack of an MMN difference between the Tone 3 and Tone 2 conditions in the current study could be that Mandarin tone 3 is underspecified (Politzer-Ahles et al., 2016) or represented as both tone 3 and sandhi tone (3V) (Li and Chen, 2015). For Politzer-Ahles et al. (2016) and Li and Chen (2015), weaker MMN effects were found when monosyllabic tone 3 served as the standard and monosyllabic tone 2 served as the deviant compared to a reverse condition. However, if Mandarin tone 3 is represented as both tone 3 and sandhi tone (3V) as Li and Chen proposed, it would be difficult to explain why there was not an MMN effect in the Sandhi condition. The activated sandhi tone (acoustically similar to tone 2) does match the surface tone of the first syllable of sandhi standards. If participants focus on the surface tone, this would lead to an MMN effect. The fact that we did not observe an MMN effect in the Sandhi condition and found comparable MMN amplitudes between Tone 2 and Tone 3 conditions may be more consistent with the underlying or even abstract (underspecified) representation view.

For the Mix condition in which standards consisted of disyllabic tone 3 sandhi words ([tone 2 tone 3] /tone 3 tone 3/) mixed with disyllabic words starting with a tone 3 syllable ([tone 3 tone 4] /tone 3 tone 4/), we did not observe an MMN effect when comparing the first syllable of standards with the deviant. Recall that this condition demonstrated a many-to-one ratio only in the underlying form between the first syllable of standards and the deviant (/tʂu2/). If Mandarin-speaking participants had paid attention to the underlying form and abstracted away from the surface variability, an MMN effect should have been observed (all standards are underlying tone 3 stimuli). The lack of an MMN effect in the Mix condition indicated that participants were not sensitive to the many-to-one ratio in the underlying form, which is necessary to elicit MMNs, or at least they could not extract the underlying many-to-one ratio to generate a significant MMN effect. The combination of tone 2 and tone 3 for the first syllable of standards on the surface, together with segmental variability in the second syllable across standards (not present in the standards in the other conditions) in the Mix condition may have made it too difficult for the participants to form any regularity for the tone of the first syllable.

### Omission MMN

An additional finding of the current study was the observation of omission MMN effects in all four conditions. These omission effects indicate a violation of participants’ prediction of hearing disyllabic stimuli, suggesting that participants did process the monosyllabic deviant as the first syllable of a disyllabic chunk. When participants did not hear the second syllable in the (monosyllabic) deviants, an omission MMN effect was generated. An omission MMN effect has been reported in the previous literature and used to suggest a predictive or rule forming mechanism in the auditory cortex (Raij et al., 1997; Yabe et al., 1997, 1998; Janata, 2001; Bendixen et al., 2009, 2014). For example, Bendixen et al. (2014) sought evidence for predictive processing from mismatch responses to omitted speech segments. In their first experiment, German words Lachs ([laks], “salmon”) or Latz ([lats], “bib”) were presented to German listeners auditorily, with La ([la], no semantic meaning) occasionally mixed in. Results showed significantly stronger omission MMN effects when the context licensed a prediction for the specific final segments (only [laks] as the standard, or only [lats] as the standard, versus [la] as the deviant) compared to when such prediction was not possible (both [laks] and [lats] as standards versus [la] as the deviant). The Bendixen et al. (2014) results show predictive coding mechanisms in the central auditory system for spoken word recognition.

The omission MMN effects observed in the current study could be interpreted in two ways. If the present omission MMNs merely reflect the absence of a second syllable, amplitudes of the omission MMNs may be predicted to be similar across conditions. However, if the current omission MMNs reflect not only the absence of a second syllable (i.e., same situation across conditions), but also predictability differences of the precise properties of the second syllable, differences in size of the omission MMNs across conditions may be expected.

The current omission MMN results showed a significantly stronger MMN effect for the Tone 2 condition than for the Tone 3 condition, Sandhi condition, and Mix condition for the multi-electrode analysis. The stronger omission MMN effect in the Tone 2 condition compared to that in the Tone 3 and Mix conditions seems to result from differences in predictability for the second syllable, suggesting either a violation of predicted segmental or tonal information of the second syllable (Näätänen et al., 1997, 2007; Bendixen et al., 2012, 2014). In the Tone 2 condition, since the deviant matched the first syllable of standards in both segmental and tonal information, participants might have deemed the deviant [tʂu2] as the beginning of the exact word used as standards (i.e., [tʂu2 je4] /tʂu2 je4/) for the Tone 2 condition. Therefore, participants in the Tone 2 condition could predict a precise second syllable for the deviant. When they did not hear the expected second syllable, a strong omission MMN effect was elicited. In contrast, for the Tone 3 condition, [tʂu3 je4] /tʂu3 je4/ was used as the standard, which mismatched the deviant in tone. Upon hearing the deviant, participants might have regarded it as the initial syllable of a different word rather than that of the standard. Consequently, neither segmental nor tonal information for the omission could be precisely predicted, leading to a weaker omission MMN effect. Regarding the Mix condition, given segmental and tonal variability on the surface, it is even more difficult for participants to predict what could have been in the omission position. This may have led to the weaker omission MMN effect observed; this condition also yielded a relatively late ERP peak (based on visual inspection). The omission MMN difference between the Tone 2 condition and Sandhi condition was also significant, which is more difficult to explain due to similar predictability of the content of the second syllable across the two conditions. Further research is required in order to investigate whether and how the Sandhi context in the standards may impact how the predictive coding of upcoming segments occurs.

### Typicality of the MMN Effects

At the onset position, MMNs emerged when the deviant and the standard differed in the F0 contour (Tone 2 condition), and when the deviant and the standard mismatched in both the underlying and the surface forms (Tone 3 condition). The MMNs elicited by the deviant in the Tone 2 and Tone 3 conditions appear to be relatively broad without a clear peak and they have a fronto-central scalp distribution, in contrast to previous studies that have observed MMNs elicited by tonal contrast which showed obvious MMN peaks (e.g., Näätänen et al., 2007; Li and Chen, 2015; Politzer-Ahles et al., 2016). However, the current MMN effects were observed in the typical 100–280 ms time window. Recall that we used disyllables as standards in all four conditions, in contrast to other studies where monosyllables were used as standards. Another unique feature of our study is that all of the deviants are expected to yield MMN not only at the onset of the first syllable, but also at the onset of the omitted second syllable. It is possible that these factors contributed to the relatively broad MMNs at the onset position in our findings. Future studies could compare the deviant response at the onset of the first syllable that are followed by omission (as in the current study) and deviant responses at the onset of the first syllable that are followed by a second syllable. This would shed light on whether the presence of a second syllable impacts the MMN waveforms.

On the other hand, the MMNs that we observed at the omission position in all four conditions show typical characteristics of the component found in previous studies (Näätänen et al., 2007; Bendixen et al., 2012, 2014). The omission MMNs emerged in the 100–280 ms time window after the onset of the omission position with an overall fronto-central scalp distribution. Unlike the broad waveforms at the onset position, the omission MMNs showed clear peaks around 100–200 ms after the onset of the omission position in the waveforms especially for the Tone 2, Sandhi, and Mix condition, although less so for the Tone 3 condition. The Tone 3 condition is the only case where the deviant and the first syllable of standards have no overlap in either surface or underlying forms. Future studies could explore this issue by testing whether the omission MMN waveforms vary depending on the similarity between the deviants and the corresponding standards across more than one level of linguistic representation.

Finally, previous studies have shown that the MMN effects elicited by durational differences between standards and deviants could be severely reduced or completely eliminated when the traditional method was employed compared to when the same-stimulus method was conducted (Shelley et al., 1991; Catts et al., 1995; Peter et al., 2010). Peter et al. (2010) explained that the reduction/absence of MMN could be due to the confound in time between the shorter deviant offset P2 and the MMN. However, the current study showed significant and typical omission MMN effects, suggesting that the omission MMN was strong enough to resist being canceled out by the P2, or the shorter deviant offset P2 and the omission MMN did not overlap in time.

In sum, the current study used disyllabic Mandarin tone 3 sandhi words to investigate how Mandarin speakers process the first syllable of tone 3 sandhi words, which is realized as a tone 2 on the surface. The present results suggest different neural mechanisms for processing canonical tone 2 syllables and sandhi tone 2 syllables in the initial position of disyllabic words. Due to the similarity between the canonical tones and the surface tones of the first syllable of standards in the Tone 2 and Tone 3 conditions, Mandarin speakers did not need to abstract away from the surface tones and process the standards. When processing tone 3 sandhi words, due to the mismatch between the canonical and surface tones, Mandarin speakers may be required to abstract away from the surface form and focus on retrieving the underlying form (Chien et al., 2016), which is either an underspecified representation or a tone 3 representation, so that they could recognize the sandhi words.

## Conclusion

The present study investigated how Mandarin tone sandhi words are processed using MMN, a cortical response allowing one to directly assess the pre-attentive processing of auditory linguistic representations. Our data suggest that different neural processing mechanisms are involved in the processing of canonical tone 2 syllables and sandhi tone 2 syllables in disyllabic sequences. We proposed two possibilities for the processing of Mandarin tone 3 sandhi words: An underspecified tonal representation assigned to the first syllable of Mandarin tone 3 sandhi words, or an underlying form /tone 3 tone 3/, both of which require Mandarin speakers to computationally convert the surface tone 2 into an underlying tone using a tone 3 sandhi rule.

## Data Availability Statement

The datasets generated for this study are available on request to the corresponding author.

## Ethics Statement

The studies involving human participants were reviewed and approved by the Human Subjects Committee at the University of Kansas. The participants provided their written informed consent to participate in this study.

## Author Contributions

Y-FC came up with the research ideas, wrote the manuscript, conducted the experiment, ran statistics, interpreted the results, and revised the manuscript. XY conducted the experiment, analyzed the data, interpreted the results, and revised the manuscript. RF discussed the research idea and methods with Y-FC, analyzed the data, interpreted the results, and revised the manuscript. JS discussed the research ideas and methods with Y-FC, analyzed the data, interpreted the results, and revised the manuscript.

## Conflict of Interest

The authors declare that the research was conducted in the absence of any commercial or financial relationships that could be construed as a potential conflict of interest.

## Appendix A

Acoustic details of the seven standard [tʂu2] tokens in the Tone 2 and Sandhi conditions, the seven standard [tʂu3] tokens in the Tone 3 condition, the four standard [tʂu2] tokens and the three standard [tʂu3] tokens in the Mix condition, and the deviant [tʂu2] for all four conditions (measured in Hz, rounded to the nearest integer).

**TABLE 1:** Tone 2 condition and Sandhi condition: Standard [tʂu2].

**Time (% of tone)**	**0%**	**10%**	**20%**	**30%**	**40%**	**50%**	**60%**	**70%**	**80%**	**90%**	**100%**	**ΔF0**	**Turning Point (ms)**
[tʂu2] (1)	160	159	157	157	160	166	173	183	194	205	208	3.19	51
[tʂu2] (2)	167	164	161	160	161	166	173	183	194	203	205	7.34	61
[tʂu2] (3)	169	164	159	157	157	160	166	176	186	194	196	12.45	71
[tʂu2] (4)	167	165	165	169	178	190	203	216	227	235	236	2.63	41
[tʂu2] (5)	164	161	157	154	154	156	159	165	173	182	186	10	81
[tʂu2] (6)	176	164	155	152	150	150	152	157	165	174	176	26.45	91
[tʂu2] (7)	155	152	149	148	149	152	157	164	174	186	191	7.44	61
Mean (SD)	165 (6.2)	161 (4.4)	158 (4.6)	157 (6.1)	158 (9.1)	163 (12.6)	169 (15.7)	178 (18)	187 (19.2)	197 (18.6)	200 (18.1)	9.93 (7.47)	65 (15.9)
													

**TABLE 2:** Tone 3 condition: Standard [tʂu3].

**Time (% of tone)**	**0%**	**10%**	**20%**	**30%**	**40%**	**50%**	**60%**	**70%**	**80%**	**90%**	**100%**
[tʂu3] (1)	158	154	147	142	138	134	131	128	126	125	125
[tʂu3] (2)	167	160	151	143	136	129	124	121	118	117	118
[tʂu3] (3)	157	154	148	142	135	131	127	126	124	124	124
[tʂu3] (4)	167	161	153	146	140	134	128	124	121	121	122
[tʂu3] (5)	176	164	153	145	138	132	127	124	122	122	122
[tʂu3] (6)	158	155	151	146	140	135	130	127	125	124	124
[tʂu3] (7)	166	160	153	147	142	138	135	132	128	126	125
Mean (SD)	164 (6.3)	158 (3.6)	151 (2.2)	144 (2.0)	138 (2.3)	133 (2.9)	129 (3.2)	126 (3.3)	124 (3.2)	123 (2.8)	123 (2.4)
											

**TABLE 3:** Mix condition: Standard [tʂu3].

**Time (% of tone)**	**0%**	**10%**	**20%**	**30%**	**40%**	**50%**	**60%**	**70%**	**80%**	**90%**	**100%**
[tʂu2] (1)	160	159	157	157	160	166	173	183	194	205	208
[tʂu2] (2)	167	164	161	160	161	166	173	183	194	203	205
[tʂu2] (5)	164	161	157	154	154	156	159	165	173	182	186
[tʂu2] (7)	155	152	149	148	149	152	157	164	174	186	191
[tʂu3] (8)	158	153	147	141	135	130	126	124	122	121	121
[tʂu3] (9)	165	156	146	138	131	126	123	121	121	122	122
[tʂu3] (10)	161	157	151	146	141	135	129	126	124	123	123
											

**TABLE 4:** Deviant [tʂu2]

**Time (% of tone)**	**0%**	**10%**	**20%**	**30%**	**40%**	**50%**	**60%**	**70%**	**80%**	**90%**	**100%**	**ΔF0**	**Turning Point (ms)**
[tʂu2] (8)	159	151	146	144	145	149	156	166	178	191	196	15.23	61
